# Oridonin loaded amphiphilic hyaluronic acid polymeric micelle with tunable redox sensitive property for CD44 targeted lung cancer therapy

**DOI:** 10.1186/s11671-026-04798-x

**Published:** 2026-07-10

**Authors:** Shaoyan Xuan, Shenglei Yang, Yuqin Mao, Zhiyong Chen, Yue Zhu, Ying Zhou, Jie Huang, Xiuliang Zhu, Zuhua Wang

**Affiliations:** 1 Department of Pharmacy, Shaoxing People’s Hospital, Zhongxing North road 568, Shaoxing, 312000 China; 2 College of Pharmaceutical Sciences, Guizhou University of Traditional Chinese Medicine, Guiyang, 550025 China; 3 Guizhou Key Laboratory of Modern Traditional Chinese Medicine Creation, Guiyang, 550025 China; 4 Traditional Chinese Medicine Preparation and Big Health Products Development-Guizhou Provincial Science and Technology Innovation Leading Talent Workstation, Guiyang, 550025 China; 5 Shaoxing Seventh People’s Hospital (Affiliated Mental Health Center, Medical College of Shaoxing University), Shengli West Road 1234, Shaoxing, 312000 China; 6 Department of radiology, The Second Affiliated Hospital, Zhejiang University School of Medicine, Hangzhou, 310000 Zhejiang PR China

**Keywords:** Oridonin, CD44 receptor, Redox-sensitive, Hyaluronic acid, Lung cancer

## Abstract

Lung cancer remains one of the most prevalent and lethal malignancies worldwide. Oridonin (ORI) exhibits remarkable anti-lung cancer activity. Nevertheless, its clinical application in lung cancer therapy is severely hindered by poor solubility, fast metabolism, insufficient tumor penetration, and low selectivity. In this study, we developed a redox-responsive polymeric micelle (HA-cys-TOS) targeting the CD44 receptor to improve ORI bioavailability and tumor targeting. The polymer was synthesized via esterification and verified by ^1^H NMR. Our data showed that ORI@HA-cys-TOS exhibited good stability, biosafety and redox-responsive sustained release. Compared to HepG2 cells, ORI@HA-cys-TOS exhibited significantly enhanced internalization ability in A549 cells, likely due to HA binding to the CD44 receptor overexpressed on A549 cells. Unexpectedly, the enhanced targeted accumulation of ORI@HA-cys-TOS micelles in vivo was also observed in both subcutaneous and in situ lung cancer tissues. In vivo and in vitro antitumor studies demonstrated that ORI@HA-cys-TOS micelles exhibited significantly greater antitumor activity compared to free ORI. Notably, no side-effects were observed during the 21 days experimental period. This study provides an experimental basis for advancing ORI toward clinical personalized lung cancer therapy by targeting CD44 and responding to the tumor redox microenvironment.

**Supplementary Information:**

The online version contains supplementary material available at 10.1186/s11671-026-04798-x.

## Introduction

Lung cancer is a rapidly growing malignant tumor that poses a significant threat to individuals’ health and lives [1]. Currently, chemotherapy and surgical treatment are the primary approaches for managing lung cancer. However, these treatments have drawbacks such as low cure rates, side effects, high recurrence rates, and the development of accompanying complications, all of which contribute to poor therapeutic outcomes. Statistical data reveals that the 5-year survival rate for lung cancer remains dismally low, at approximately 15% [2]. As lung cancer advances to its intermediate and late stages, surgical treatment is often deemed unsuitable due to the elevated risk of metastasis spread. To date, a wide range of chemotherapeutic agents have been utilized in lung cancer treatment. However, the efficacy and overall success of anticancer therapies are significantly compromised by toxicity associated with chemotherapy and the emergence of acquired therapeutic drug resistance [3–4].

Oridonin (ORI), an ent-kaurane diterpenoid isolated from the traditional Chinese medical plant *Rabdosia rubescens* and its related species, exhibits potent antitumor activity against a broad spectrum of cancer cells. Owing to its favorable anticancer properties, ORI has attracted growing research interest. Accumulating studies have also proven that this natural compound can effectively improve the survival outcomes of tumor patients [5]. It exhibits inhibitory effects on various types of cancers, including lung cancer, breast cancer, cervical cancer, and others [6–8]. ORI primarily exerts an anti-tumor effect through mechanisms such as inhibiting the cell cycle, reducing telomerase activity, inducing apoptosis in tumor cells, and reversing drug resistance [9–10]. The growing body of evidence suggests that ORI holds promise as a potential clinical antineoplastic drug. However, oridonin exhibits low solubility in water (0.75 g/L at 37 °C) and limited drug dissolution. It also has a low bioavailability (only 10% absolute oral bioavailability) and is unstable in water, making it susceptible to degradation. These shortcomings greatly restrict its clinical application [11].

In recent decades, the development and implementation of nano-drug delivery systems have partially addressed the challenges encountered in tumor drug therapy, such as limited bioavailability and insufficient drug accessibility. Currently, the most prevalent nano-drug delivery systems include liposomes, polymer micelles, nanoparticles, and others [12]. Among these versatile nanocarriers, polymer micelles are widely recognized as a highly promising drug carrier system [13]. Hyaluronic acid ( HA) is a naturally occurring glycosaminoglycan found throughout the body. It is recognized for its biocompatibility, biodegradability, and intrinsic capacity for natural degradation [14]. HA is also a key component of the extracellular matrix, contributing to diverse various cellular processes including tissue remodeling, intercellular space expansion, inflammation and tumorigenesis [15]. CD44, the primary HA receptor and main site for HA binding on cells, is a widely expressed cell surface protein that faciliate cell adhesion to extracellular matrix components or specific cell surface ligands in many tumors. Research indicates that the binding of HA to CD44 receptors on the surface of tumor cells has implications for tumor growth and metastasis, facilitates the targeted internalization of hyaluronic acid-based nano-drug delivery systems into A549 tumor cells with high expression of CD44 receptors [16, 17]. Alpha-tocopherol succinate (ɑ-TOS) is the main vitamin E compound in humans, which has been used as an immune supplement and possesses important antioxidative and immunomodulatory properties. Numerous studies have shown that TOS has potential as an anti-tumor agent, as it can inhibit the proliferation of various tumor cells both in vivo and in vitro, without significantly affecting normal cells [18]. Additionally, vitamin E derivatives possess several fundamental functions for drug delivery applications, including enhanced stability, hydrophobicity, and water-solubility [19].

In this study, the tumor targeted chemotherapy nano-platform is used for the treatment of lung cancer. ORI-loaded nanoparticles are designed to target tumor cells through CD44 receptors, ensuring the specific delivery of nanoparticles and enhancing treatment safety. Additionally, the high concentration of glutathione in tumor cells triggers the breakdown of polymer micelle molecules, resulting in the release of the antineoplastic drug ORI [20]. This mechanism enhances the efficacy of chemotherapy and further contributes to safer and more effective tumor-targeted chemotherapy. This study systematically investigated the in vitro drug release, cell targeted uptake, cytotoxicity, in vivo distribution, in vivo toxicity, and anti-tumor effects of HA-cys-TOS-loaded ORI polymer targeted immune nanoparticles.

This study aims to enhance the bioavailability and stability of oridonin and evaluate its efficacy in targeted treatment of lung cancer. The findings pave the way for developing innovative antineoplastic drugs and formulations, advancing clinical strategies for targeted chemotherapy in lung cancer. This approach is expected to accelerate the clinical application of oridonin, ultimately improving the survival rate and treatment outcomes for cancer patients.

## Materials and methods

### Materials

Hyaluronic acid (HA) was purchased from Shanghai Ian Chemical Technology Co., Ltd. (Shanghai, China). N-hydroxysuccinimide (NHS) was purchased from Sahn Chemical Technology Co., Ltd. (Shanghai, China). Cystamine dihydrochloride (cys), 1-ethyl-(3-dimethylaminopropyl) carbodiimide hydrochloride (EDC), Oridonin, ɑ-tocopherol succinate (TOS), Reduced glutathione and Coumarin 6 were purchased from Shanghai Aladdin biochemical Technology Co., Ltd. (Shanghai, China). Reduced glutathione detection kit and 0.25%Trypsin-EDTA trypsin were purchased from Beijing Solebao Technology Co., Ltd. (Beijing, China). RPMI.1640 and PBS medium were purchased from Zhejiang Senrui Biotechnology Co., Ltd. (Zhejiang, China). Fetal Bovine Serum was purchased from Zhejiang Tianhang Biotechnology Co., Ltd. (Zhejiang, China). Penicillin streptomycin mixture was purchased from GIBCO Co., Ltd. (New York, USA). CD31 was purchased from Wuhan Sanying Biology Technology Co., Ltd. (Wuhan, China). CD44 was purchased from ABclonal Technology Co., Ltd. (Wuhan, China) and CCK-8 was purchased from APExBIO Technology Co., Ltd. (Houston, USA)

### Cell culture

The cell lines in this study, A549 cells (CCL-185, ATCC) and HepG2 cells (CRL-3581, ATCC), were generously provided by the College of Pharmaceutical Sciences, Zhejiang University. These cells were cultured in RPMI-1640, 10% FBS and 1% penicillin-streptomycin. The culture conditions involved maintaining the cells at a temperature of 37 °C with a 5% CO2 atmosphere.

### Preparation of HA-cys-TOS polymer

HA-cys-TOS polymer was synthesized using a two-step amidation reaction according to a previously reported method [21]. HA-cys was synthesized through the amidation reaction between the carboxyl group at one end of HA and the amino group at the other end of cysteine hydrochloride. Briefly, 2.0 g of Hyaluronic acid (HA, 10000 Da) was dissolved completely in 100 mL of PBS. Subsequently, a mixture containing 0.46 g of N-hydroxysuccinimide (NHS) and 0.77 g of 1-(3-dimethylaminopropyl)-3-ethyl carbodiimide (EDC) was added to the solution. The mixture was then stirred at 25 °C for 1 h for reaction. Following that, 1.80 g of cys was added to the mixture, and the reaction was allowed to proceed for 4 h. After completion of the reaction, the solution was transferred into a dialysis bag (Molecular weight cut-off, MWCO 3500) and dialyzed against deionized water for 48 h. The dialysed liquid was passed through 0.45 μm filter membrane and freeze-dried to obtain the intermediate HA-cys.

HA-cys-TOS was synthesized by amidation reaction between the terminal carboxyl group of ɑ-tocopherol succinate (TOS) and the terminal amino group of HA-cys. Briefly, 2.12 g of TOS was stirred and dissolved in 200 mL of anhydrous ethanol. Then, 2.30 g of NHS and 3.83 g of EDC were added to activate the carboxyl group of TOS at a temperature of 25 °C for a duration of 1 h. Simultaneously, 2.0 g of intermediate HA-cys (~ 14000 Da) was stirred in pure 200 mL ultra-pure water until it completely dissolved. Subsequently, the TOS reaction solution was slowly dripped into the solution at 25 °C for a period of 24 h. Following this, the reaction solution was poured into 4 L of ice anhydrous ethanol, and the resulting precipitate was re-dissolved in ultra-pure water through centrifugation at 3800 × g and passed through a 0.45 μm water film. Finally, the sample was freeze-dried to obtain HA-cys-TOS. The structure of HA-cys-TOS was analyzed using 1 H-NMR spectroscopy technique.

### Preparation and characterization of ORI@HA-cys-TOS micelles

An appropriate amount of HA-cys-TOS was dissolved in ultra-pure water to prepare a micellar solution with a concentration of 10 mg/mL, then added enough ORI anhydrous ethanol solution (1 mg/mL) to the micellar solution. The mixture was stirred at room temperature in a brown sample bottle for 24 h. After that, the mixture was dialyzed against DI water in a dialysis bag (MWCO: 3.5 kDa) for 4 h to effectively remove impurities. Finally, the solution was freeze-dried through a 0.45 μm microporous filter membrane to obtain the ORI-loaded micelles ORI@HA-cys-TOS. The amount of ORI in the micelles was determined using high performance liquid chromatography (HPLC, Agilent, China), and the specific HPLC chromatographic conditions were set as follows: WondaSil C18 chromatographic column; mobile phase consisting of methanol and ultra-pure water (54:46, v/v); flow rate of 1.0 mL/min; detection wavelength of 240 nm; injection volume of 20 µL; and column temperature maintained at 25 °C. The detailed HPLC detection method was further supplemented in the Supplementary Material. The encapsulation efficiency (EE) and drug loading (DL) capacity of ORI@HA-cys-TOS were calculated according to following formulas (1) and (2).

1 $$ EE\left( \% \right) = C1 \times V/W1 \times 100\% $$

2 $$ DL\left( \% \right) = C1 \times V/\left( {C1 \times V + W2} \right) \times 100\% $$

where C1 is the concentration of ORI in methanol (µg/mL), V refers to the volume of methanol (mL) added when ORI@HA-cys-TOS micelle is destroyed, W1 and W2 represent the mass of ORI (mg) added and the total mass of HA-cys-TOS micelles (mg) added, respectively.

The particle size, polydispersity index (PDI) and zeta potential of ORI@HA-cys-TOS were detected by a DelsaMaxPRO Beckman nanoparticle size analyzer (Beckman, USA). Prior to detection, the freeze-dried micelle powder was redispersed in ultra-pure water to form a uniform aqueous solution (0.1 mg/mL), and the measurement was performed at room temperature to obtain the particle size distribution and PDI values reflecting micelle dispersion uniformity.

The microscopic morphology and structural characteristics of ORI@HA-cys-TOS micelles were observed by transmission electron microscopy (TEM; JEOL JEM-1230, Japan). For TEM sample preparation, a drop of diluted uniform micelle aqueous solution was dripped onto a copper grid, dried naturally at room temperature, and then observed and photographed under the transmission electron microscope to analyze the morphological integrity and structural uniformity of the micelles.

### Determination of critical micelle concentration

The critical micelle concentration (CMC) of HA-cys-TOS polymer was determined by fluorescence spectrometry, as described in the literature [22]. Briefly, a pyrene solution with a concentration of 1.2 × 10^−5^ mmol / mL was prepared with methanol and placed in a brown volumetric flask, and the methanol was quickly evaporated by nitrogen. Various concentrations of micelle solution (1.953–500 µg/mL) were added to a pyrene methanol solution. The solution was then subjected to ultrasonication for 30 min in a water bath. After that, it was balanced at 37 °C in a constant temperature water bath oscillator at a speed of 10 × g for 24 h. The emission spectra of the micellar solutions containing pyrene were scanned at wavelengths ranging from 300 to 500 nm, with an excitation wavelength of 334 nm and an excitation slit of 5 nm. The ratio of the emission spectra at 374 nm and 385 nm, denoted as I374/I385, was calculated. The concentration corresponding to the intersection of the fitting curve, obtained by plotting the logarithm of concentration (LogC) against I374/I385, was determined as the critical micelle concentration (CMC).

### Hemolysis test

Targeted preparations are frequently administered through intravenous injection. Red blood cell was extracted from fresh rat whole blood and mixed with PBS to prepare 2% red blood cell suspension for hemolysis experiment to investigate the biocompatibility of micelles during transport in vivo [23–24]. 40 mg of ORI@HA-cys-TOS micelles were accurately weighed and cold saline was used to prepare a 4 mg/mL solution, which was then diluted to various concentrations. The micellar solution of different concentrations (1.5 mL each) and an equal amount of red blood cell suspension were added to the 5mL EP tube. The mixture was shaken at 10 × g for 30 min in a constant temperature concussion box at 37 °C. After centrifugation for 15 min in a 96-well plate, 100 µL of the supernatant was collected and the absorbance at 540 nm was measured using an enzyme labeling instrument. Physiological saline was used as a negative control group, and distilled water was used as a positive control group. The percentage of hemolysis was calculated by applying the following formula (3).

3 $$ {\mathrm{Hemolysis}}\left( \% \right) = \left( {{\mathrm{A}}_{{{\mathrm{sample}}}} - {\mathrm{A}}_{{{\mathrm{negative}}}} } \right)/\left( {{\mathrm{A}}_{{{\mathrm{positive}}}} - {\mathrm{A}}_{{{\mathrm{negative}}}} } \right) \times 100\% $$

where A_sample_, A_negative_ and A_positive_ are the absorbance values of ORI@HA-cys-TOS micelles, physiological saline and distilled water at 540 nm measured by enzyme labeling instrument, respectively.

### In vitro drug release behavior study

The release behavior of HA-cys-TOS micelles was studied in vitro using dialysis. A dialysis bag with a molecular weight cutoff of 3500 Da was filled with 2 mL of the drug and immersed in a release medium consisting of 30mL of phosphate buffer. The bag was then placed in a shaker and incubated at 37 °C. At specific time intervals (0.5, 1, 2, 4, 8, 12, 24 h), 1mL of dialysate solution was collected and an equal volume of release medium was added. The drug content in the release medium was measured using HPLC instrument to calculate the cumulative drug release rate. Each experiment was performed in triplicate.

### CD44 receptor expression

The culture medium in the culture bottle containing A549 and HepG2 cells was poured out and washed twice with pre-cooled PBS. After removing the liquid, 200 µL of RIPA lysate was added to each bottle and the cells were incubated on ice for 5 min. The lysate was then collected by centrifuging at 12,000 × g to obtain the supernatant. The protein extract (50 µg) was separated using 10% SDS-PAGE electrophoresis and transferred onto a 0.45 μm PVDF membrane. During room temperature incubation (RT), the protein was blotted with the first antibody (anti-CD44 antibody, ABclonal Technology) for 6 h. Subsequently, the goat anti-mouse IgG antibody (1:5000) was used for anti-CD44 antibody detection, with incubated for 2 h at RT.

### Effect of ORI on tumor microenvironment

This experiment aimed to investigate the effect of ORI on the tumor microenvironment. Glutathione at a concentration of 1 mg/mL was prepared using distilled water. Different concentrations of oridonin (500, 250, 125, 62.5 µg/mL) were added to a 2 mL EP tube to ensure full contact between oridonin and GSH. The mixture was allowed to incubate for 1 min. After being placed at room temperature for 24 h, the GSH content was determined using the reduced glutathione test box.

### Cellular uptake study

The cellular uptake behavior of ORI@HA-cys-TOSmicelles was investigated in vitro using coumarin 6, a fat-soluble compound with green fluorescence [25]. ORI@HA-cys-TOS micelles, labeled with coumarin 6, were prepared through dialysis method. A549 and HepG2 cells were seeded on 24-well plates at a density of 2 × 10^4^ cells/well and incubated for 12 h to allow complete adhesion. Subsequently, the cells were incubated with a fluorescence-labeled ORI@HA-cys-TOS solution for varying durations of 4, 2, and 1 h. Afterward, the cells were washed twice with preheated PBS, and the fluorescence distribution within the cells was observed using an inverted microscope. (Leica DMIL LED; Leica, Solms, Germany)

### In vitro cellular competitive uptake studies

To investigate the tumor targeting of ORI@HA-cys-TOS micelles, A549 cells were stained with the fluorescent membrane intercalating dye, PKH67 [26]. Briefly, A549 cells were collected, re-suspended, and washed with serum-free culture medium. After centrifugation, the cells were dispersed in 200 µL of diluent C. Then, 200 µL of diluent C containing trace PKH67 fluorescent dye was added to the cell mass and quickly mixed. The mixture was incubated at room temperature without light for 10 min. At the end of the incubation period, 1.5 mL of serum was added and incubated for 2 min to stop the staining process. The mixture was then centrifuged for 10 min at 110 × g. After centrifugation, the cells were washed with 10 mL of culture medium and centrifuged for 8 min. This washing step was repeated three times before the cells were set aside. Prior to the experiment, 10 mm aseptic glass plates were prepared. PKH67-stained A549 cells and unstained HepG2 cells were co-inoculated into 24-well plates at a total density of 2 × 10^4^ cells/well. The cells were maintained in a culture medium consisting of 1640 and MEM (1:1) to ensure cell viability. The A549/HepG2 co-incubation model was created by allowing the cells to incubate in the incubator for 12 h.

Following the growth and adhesion of A549/HepG2 co-incubated model cells, a specific quantity of fluorescence-labeled ORI@HA-cys-TOS solution was added and incubated for 2 h. Additionally, DAPI was utilized for nuclear staining, with an incubation period of 0.5 h. Subsequently, any excess dyes were removed by washing with an appropriate amount of PBS, and the cells were fixed with 4% paraformaldehyde for a duration of 10 min. The slides were fixed with nail polish, and then 10 µL of anti-fluorescence quenching solution was added. The absorption and distribution of A549 cells and HepG2 cells in the co-culture system were observed under a fluorescence microscope.

### In vivo tumor-targeting studies

Female SPF-grade BALB/c-nu nude mice at 5 weeks of age were purchased from SPF (Beijing) Biotechnology Co., Ltd., with the animal license number SCXK (Beijing) 2019-0010. All animal studies were conducted in accordance with the Guidelines for the Care and Use of Laboratory Animals, which was approved by the Animal Experimental Ethics Committee of Guizhou University of Traditional Chinese Medicine (Grant No.20220053). The in vivo bio-distribution of ORI@HA-cys-TOS micelles was further evaluated in different lung cancer models (subcutaneous and in-situ). Briefly, DIR (a near infrared fluorescent fuel) labeled ORI@HA-cys-TOS micelles were firstly prepared by dialysis method, then intravenously injected into A549 tumor-bearing mice [27]. The mice were observed by the IVIS Lumina II in vivo imaging system (Caliper Life Science, USA) at the predetermined time (1, 2, 4, 8, 12, 24 and 48 h) after the injection. Then, mice mice were deeply anesthetized with 3.0%–3.5% isoflurane in medical oxygen until loss of consciousness (confirmed by absence of corneal reflex, respiratory movement and tail pinch response), followed by cervical dislocation for humane euthanasia. Various tissues, including tumors, were collected, weighted and observed by the in vivo imaging system. The accumulation of ORI@HA-cys-TOS micelles in various tissues was calculated as %ID/g (the percentage of the injected dose per gram of tissue).

### Immunofluorescence staining analysis

The tumor tissue sections in A549 and HePG2 tumor model were stained with FITC-labeled CD31 and CD44 antibodies to observe the distribution of the micelles in tumor vessels and the expression level of the tumor CD44 receptor [28]. Additionally, the nucleus was re-stained with DAPI and photographed using a fluorescence microscope.

### In vitro cytotoxicity study

The cytotoxicity of free ORI, HA-cys-TOS and ORI@HA-cys-TOS were assessed using the CCK-8 method [29]. A549 cells were seeded in 96-well plates at a density of 4.5 × 10^3^ cells per well and incubated at 37 °C in a 5% CO2 incubator. After 24 h of cell adhesion, drugs were added. Untreated cells served as the negative control, while ORI was used as the positive control group. Each concentration was tested in parallel five times. After 24 h of incubation, 10 µL of CCK-8 solution was added to each well and incubated for 2 h. The absorbance of cells at 450 nm was measured using an enzyme labeling instrument to calculate the cell survival rate. The cell survival rate (cell viability) was calculated by the following formula (4):

4 $$ {\mathrm{Cell}}\;{\mathrm{viability}}\left( \% \right) = \left( {{\mathrm{A}}_{{\mathrm{s}}} - {\mathrm{A}}_{{\mathrm{b}}} } \right)/\left( {{\mathrm{A}}_{{\mathrm{c}}} - {\mathrm{A}}_{{\mathrm{b}}} } \right) \times 100\% $$

where A_s_ is the absorbance of cells treated with different formulations, A_c_ is the absorbance of untreated control cells, and A_b_ corresponds to the absorbance of blank medium without cells.

### In vivo antitumor activity studies

A549 cells were collected, re-suspended in PBS and injected subcutaneously into the right hind leg of BALB/C nude mice at a density of 5 × 10^7^ cells to establish the subcutaneous lung cancer model [30]. Female BALB/C nude mice bearing A549 tumors were randomly divided into 3 groups (*n* = 6), including saline, free ORI and ORI@HA-cys-TOS groups. The mice were intravenously injected with saline, free ORI (10 mg ORI/kg) and ORI@HA-cys-TOS (10 mg ORI/kg). The tumor size of each mouse was measured daily using a vernier caliper, while the body weight of mice bearing BALB/C-nu tumor was also recorded. The experiment was terminated on day 21 after initial treatment. After 21 days of treatment, the orbital blood of every group mice was collected to obtain serum by centrifuging at 950 × g for 20 min for biochemical analysis, then the mice were sacrificed (For details on mouse euthanasia, see 2.12). The tumor and main organs were collected for further Ki67 and H&E staining.

### Statistical analysis

Student’s T-test was used to determine the statistical significance of differences between groups. For all analyses, statistical significance was set at *P* < 0.05 for each paired experiment.

## Results

### Synthesis and characterization of HA-cys-TOS polymer

**Scheme 1:**
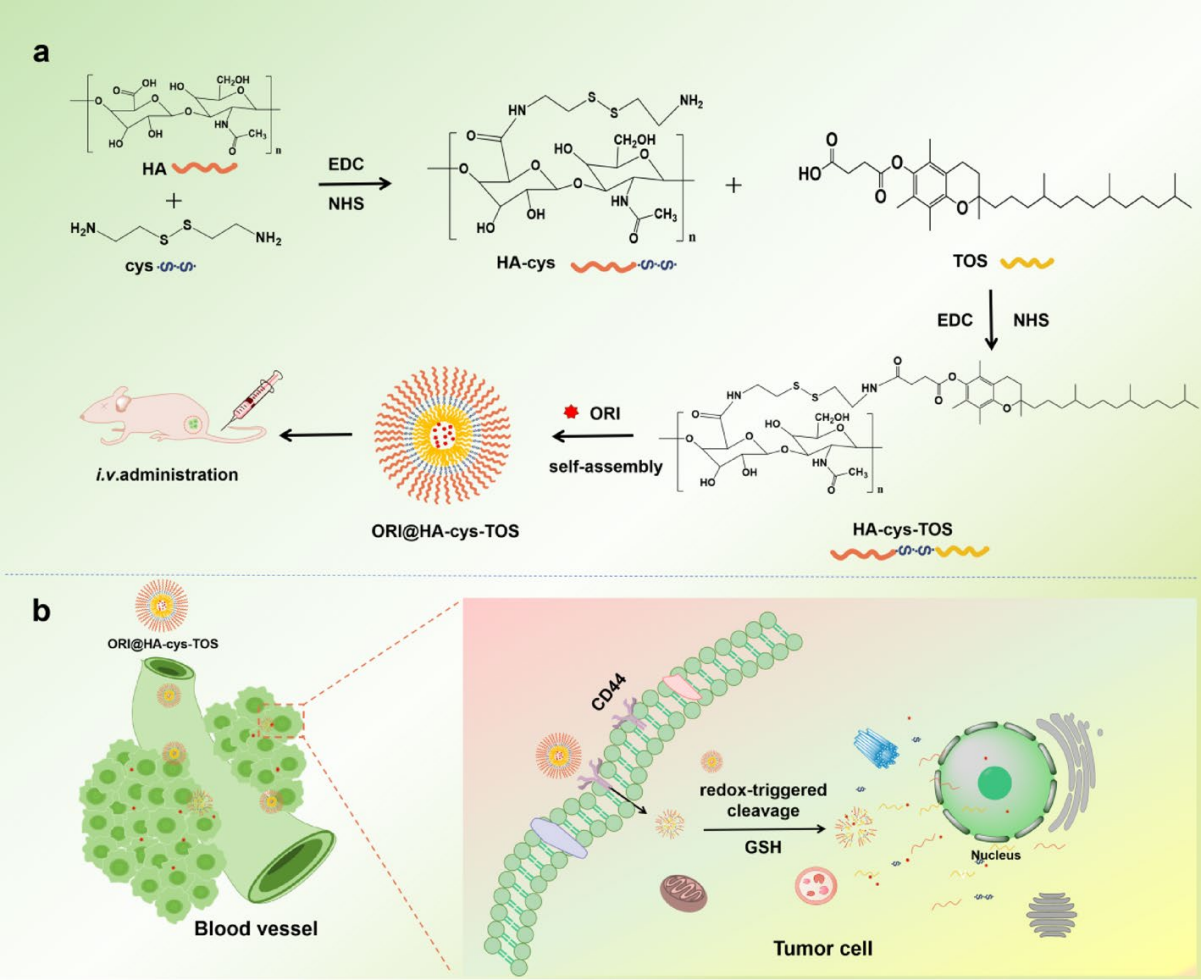
Schematic illustration of the delivery of oridonin by HA-cys-TOS polymer drug-loaded nano-micelles. **a** Synthesis and self-assembly of ORI loaded HA-cys-TOS polymers; **b** The targeted delivery ORI@HA-cys-TOS micelles into tumors mediated by CD44 receptors

To enhance the stability and bioavailability of ORI, we developed a ROS-sensitive and CD44 receptor-targeting nano-drug delivery system-based HA and ɑ-TOS (Scheme 1). The delivery system employs cystamine as a connector to conjugate HA (a hydrophilic main chain) with ɑ-TOS (a hydrophobic fragment) through an amidation reaction. This results in the formation of hyaluronic acid-cystamine-ɑ-tocopherol succinate (HA-cys-TOS) polymer micelles. We hypothesized that the chemotherapy effect of ORI, facilitated by HA-cys-TOS polymer micelles, could selectively deliver ORI into the tumor cells and regulate its release within the cellular redox microenvironment, thereby enhancing the micelles’ antitumor efficacy.

**Fig. 1:**
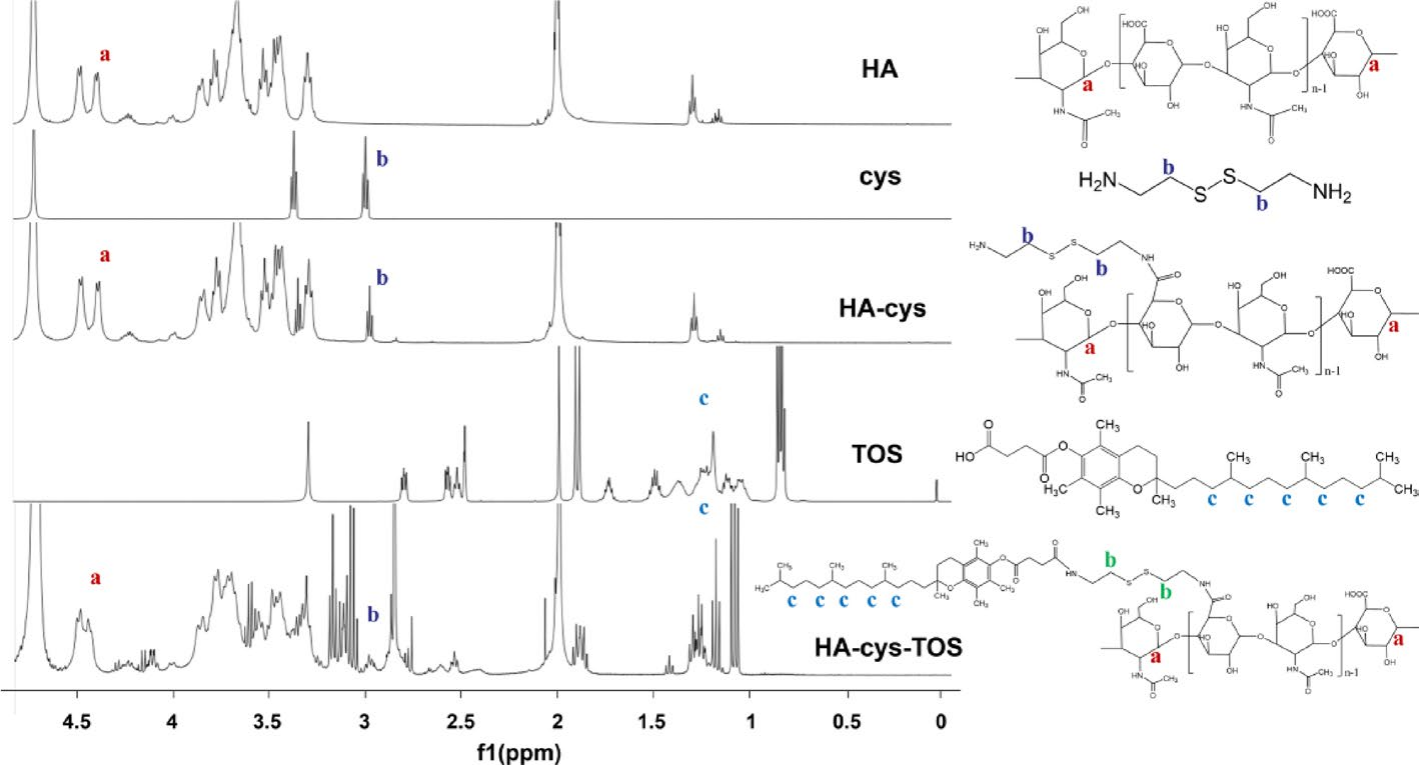
^1^H NMR spectra of HA, cys, HA-cys, TOS and HA-cys-TOS from top to bottom, respectively

HA-cys-TOS polymer was synthesized through an amidation reaction and confirmed using 1NMR. Figure 1 shows the HA-cys-TOS nuclear magnetic spectrum, with the deuterated water solvent peak serving as the internal standard. The peak (a) at δ: 4.55-4.45ppm represents the characteristic peak of the sugar ring hydrogen of hyaluronic acid. Peak b (δ: 3.03 ppm) corresponds to the characteristic peak of cystamine, while peak c (δ: 3.03 ppm) represents the proton peak of the carbon chain methyl hydrogen on ɑ-tocopherol succinate. The presence of characteristic peaks a and b in the hydrogen spectrum of HA-cys suggests the successful synthesis of intermediates. Additionally, the characteristic peaks of HA, cys, and TOS were observed in the 1 H-NMR spectrum of HA-cys-TOS. These results indicate the successful synthesis of the polymer micelle HA-cys-TOS, which was achieved by bridging hydrophilic hyaluronic acid and hydrophobic ɑ-tocopherol succinate using cystamine.

**Fig. 2:**
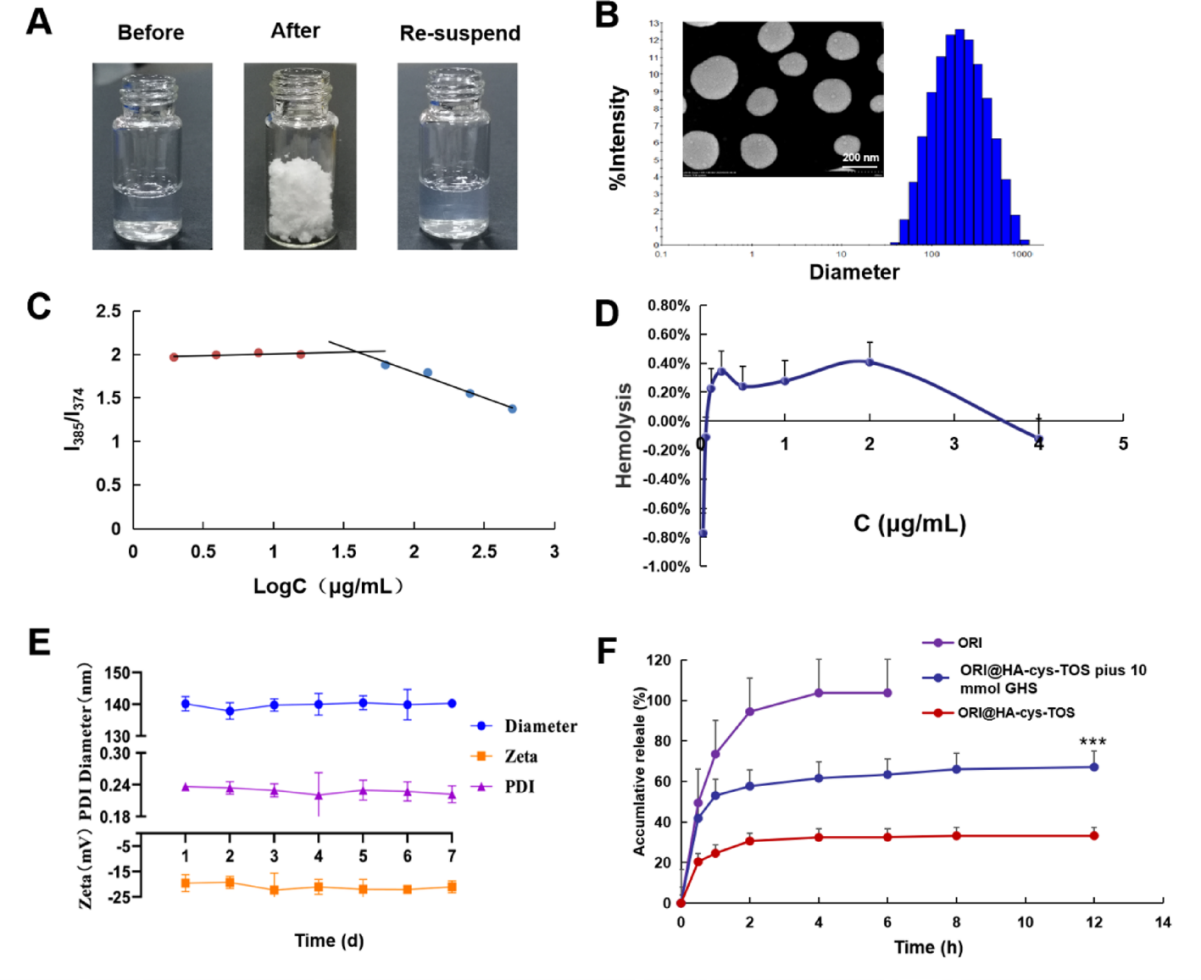
Characterization of ORI@HA-cys-TOS. **A** The pictures for the solution containing ORI@HA-cys-TOS micelles (before lyophilization), the powder of ORI@HA-cys-TOS micelles (after lyophilization) and the solution containing ORI@HA-cys-TOS micelles (the re-suspended). **B** Particle size distribution and TEM image of ORI@HA-cys-TOS micelles. **C** Variation of intensity ratio (I_374_/I_385_) vs. concentration of HA-cys-TOS. **D** The hemolysis rate of ORI@HA-cys-TOS. **E** Stability testing of ORI@HA-cys-TOS micelles in PBS during 7-day storage at room temperature. **F** GSH-triggered ORI release from ORI@HA-cys-TOS micelles for 12 h. Data are presented mean ± SD (*n* = 3, ***P < 0.001)

The critical micelle concentration (CMC) of HA-cys-TOS in aqueous media was measured by fluorometry, as our previously reported [31]. CMC value of HA-cys-TOS polymer in distilled water was about 39.3 µg/mL (Fig. 2C), which indicated that HA-cys-TOS has excellent dispersity and ability to self-assemble into small nanomicelles in an aqueous environment.

### Preparation and characterization of ORI@HA-cys-TOS micelles

ORI loaded HA-cys-TOS (ORI@HA-cys-TOS) micelles were successfully prepared by dialysis method. The encapsulating efficiency (EE) and drug loading (DL) of the micelles were determined using HPLC. The ORI content determination method established in this study was shown to be accurate and reliable, as demonstrated by the validation results of the specificity (Figure S1), linearity (Figure S2), precision (Table S1), repeatability (Table S2), stability (Table S3) and recovery rate (Table S4).

**Table 1:** Particle size, PDI and Zeta potential of HA-cys-TOS and ORI@HA-cys-TOS

Sample	Diameter (nm)	PDI (-)	Zeta potential (mV)	EE (%)	DL (%)
HA-cys-TOS	140.2 ± 0.9	0.237 ± 0.003	− 19.56 ± 1.34	–	–
ORI@HA-cys-TOS	162.8 ± 2.2	0.243 ± 0.004	− 22.49 ± 0.75	96.73 ± 0.02	6.75 ± 0.16

Data represent the mean±standard deviation (*n* = 3)

Table 1 showed the ORI encapsulating efficiency (EE) and drug loading (DL) of ORI@HA-cys-TOS micelles. The average particle size of HA-cys-TOS micelles was 140.2 ± 0.9 nm, determined by DLS. Due to the encapsulation of ORI, the average particle size of ORI@HA-cys-TOS micelles increased to be 162.8 ± 2.2 nm, determined by DLS, which was generally consistent with the TEM value (about 170 nm, Fig. 2B). The slight discrepancy is likely attributed to minor particle aggregation during sample drying. ORI encapsulating efficiency of ORI@HA-cys-TOS was about 97%, which indicated that HA-cys-TOS could efficiently load ORI into the micelles. It was also found that lyophilized ORI@HA-cys-TOS powder could easily disperse in the deionized water to formulate the micelles again, and there were no significant particle size change, compared with the micelles before the lyophilization (Fig. 2A). Hemolysis is a crucial initial assessment parameter for determining the toxicity of biomaterials. Figure 2D showed that the hemolysis rate of ORI@HA-cys-TOS was below 5%, indicating excellent blood compatibility and compliance with medical device standards. The stability of ORI@HA-cys-TOS micelles stored at room temperature for 7 days was presented in Fig. 2E. The micelles have a particle size of approximately 140 nm, a dispersion index PDI of around 0.2, and a Zeta potential of approximately − 20 mV. These findings demonstrated that the micelles have favorable stability over a 7-day period, facilitating short-term storage and transportation. These results suggested that ORI@HA-cys-TOS micelles have good physical-chemical stability, the intravenous injection for the administration is safe.

Figure 2F shows the behaviour of ORI release from ORI@HA-cys-TOS micelles. It was crystal clear that almost all of free ORI was released within 6 h. In order to investigate the stimuli-responsive release properties of ORI@HA-cys-TOS micelles, the ORI release of the micelles solutions with and without GSH (10mmol/mL) was synchronously evaluated. The results showed that a rapid ORI increase from ORI@HA-cys-TOS (with GSH) micelles was observed during the following 12 h period with a release amount of 67.12%, when the released ORI diffused through the dialysis membrane into the external medium. However, ORI release from the ORI@HA-cys-TOS (without GSH) micelles was slow significantly (*P* < 0.001), with only about 30% ORI accumulative release after 12 h, compared with ORI@HA-cys-TOS (with GSH) group, which validated the stimuli-responsive release potential of ORI@HA-cys-TOS. The results suggested that it is feasible to utilize the tumor microenvironment to trigger the redox-responsive release of ORI. This phenomenon has the potential to enhance the therapeutic efficacy of chemotherapeutic drugs.

### Cell uptake of ORI@HA-cys-TOS micelles

**Fig. 3:**
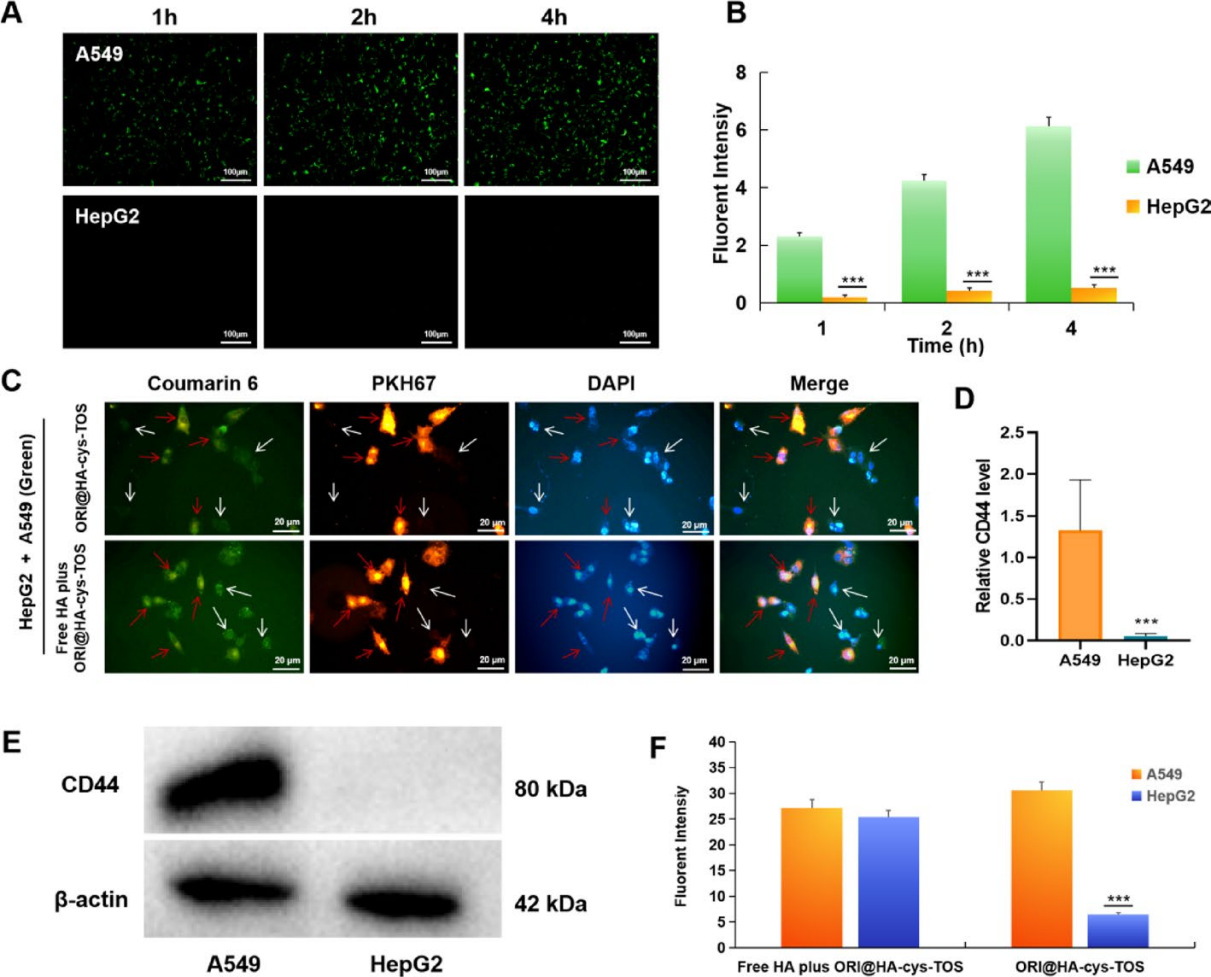
Cellular uptake studies. **A** Cell uptake of ORI@HA-cys-TOS (coumarin 6 labeled) in A549 and HepG2 cells with different incubation time. **B** The quantitative analysis based on the imaging in (A) by a software “Image J”. **C** Cellular competitive uptake images of coumarin 6 labeled micelles after 2 h of incubation. All cells were all stained with DAPI. A549 cells were labeled with PKH67 fluorescent linker (Green), and co-cultured with HepG2 cells. Then, the cells were incubated with ORI@HA-cys-TOS and ORI@HA-cys-TOS plus free HA. **D** The quantitative analysis based on the imaging in (E) by a software “Image J”. **E** Western blot analysis of CD44 expression in A549 and HepG2 cells. **F** The quantitative analysis based on the imaging in (C) by a software “Image J”. All data represented the mean ± standard deviation (*n* = 3, *P < 0.05, **P < 0.01, ***P < 0.001)

The amount of CD44 present in lung cancer cells determines the targeting ability of HA-cys-TOS micelles. Western blotting analysis confirmed high expression levels of CD44 receptor in A549 cells, but only low expression level in HepG2 cells (Fig. 3D and E and S3). As shown in Fig. 3A, ORI@HA-cys-TOS micelles exhibited a significantly higher cellular internalization in A549 cells with high CD44 expression than in HepG2 cells with low CD44 expression throughout the entire 4 h incubation period, as quantitatively confirmed by Image J software (Fig. 3B). The targeting property of ORI@HA-cys-TOS micelles was further detected by co-incubating A549 with HepG2 cells (A549/HepG2). Figure 3C and F further illustrated that the fluorescence intensity of CD44-positive A549 cells (red arrows) was notably stronger compared to that of CD44-negative HepG2 cells (white arrows). In the blocking experiments, A549/HepG2 cells were incubated firstly with free HA (40 mg/mL) for 0.5 h and then with ORI@HA-cys-TOS micelles. A549 cell uptake of ORI@HA-cys-TOS micelles obviously decreased due to the addition of free HA. These findings suggested that HA could specifically bind to CD44 receptor-positive A549 cells, indicating the potential of ORI@HA-cys-TOS micelles for targeting lung cancer cells.

### In vivo tumor-targeting studies

**Fig. 4:**
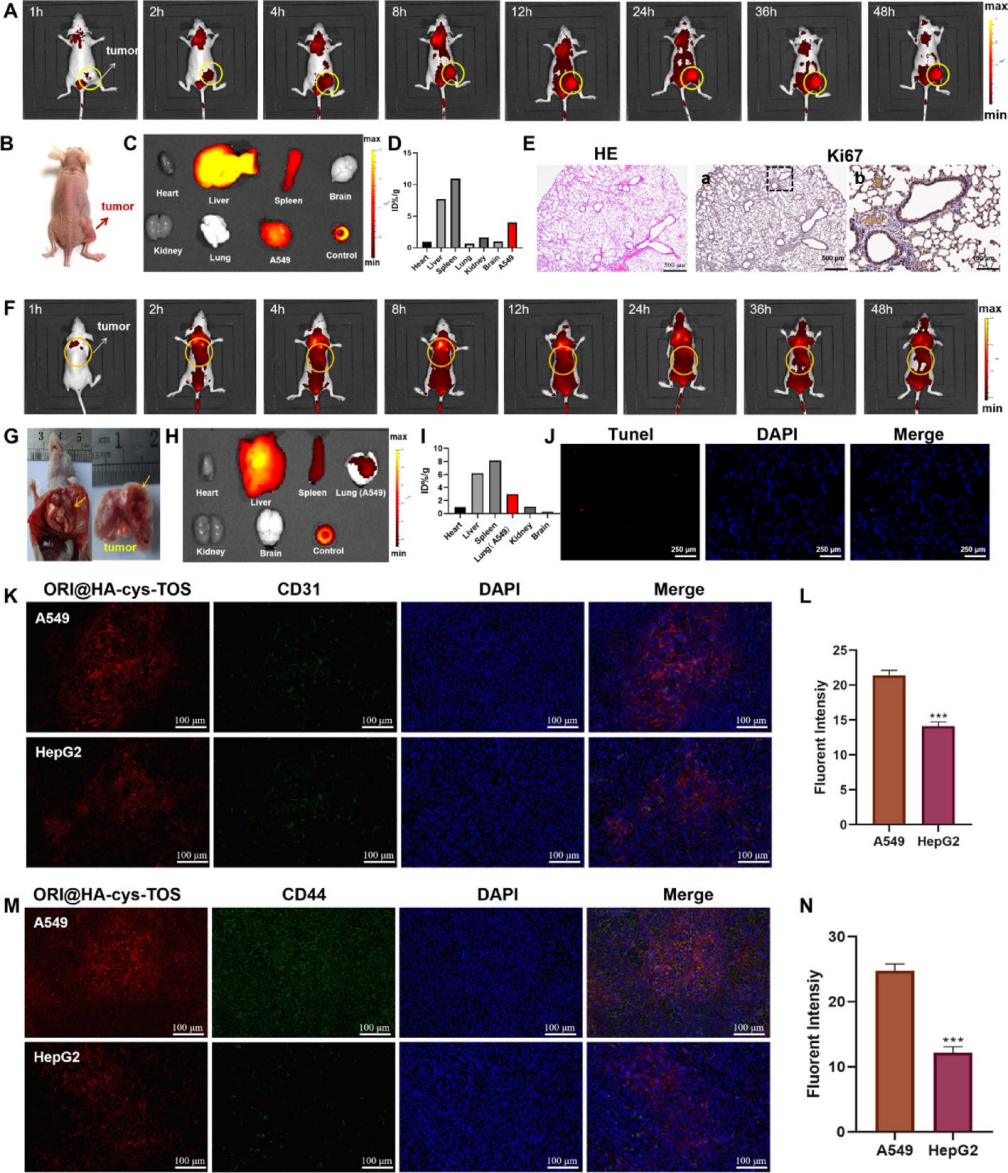
The in vivo tumor-targeting studies. **A** The in vivo imaging of the mice, bearing subcutaneous A549 tumors, at different time after iv injection of ORI@HA-cys-TOS micelles encapsulating DIR. **B** The picture of subcutaneous A549 tumor bearing BALB/C-nu mice. **C** The fluorescent imaging of various tissues in subcutaneous tumors model at 48 h after the iv injection of the micelles. **D** The accumulation of ORI@HA-cys-TOS micelles in various tissues of (C) was calculated as %ID/g (the percentage of the injected dose per gram of tissue). **E** H&E and Ki67 staining results of lung tissue in in situ A549 tumor model of BALB/C-nu mice (40×). **F** The in vivo imaging of ORI@HA-cys-TOS micelles in in situ A549 tumors at different time after iv injection. **G** The photograph of in situ lung cancer in BALB/C-nu mice. **H** The fluorescent imaging of various tissues in in situ tumors model at 48 h after the iv injection of the micelles. **I** The accumulation of ORI@HA-cys-TOS micelles in various tissues of (H) was calculated as %ID/g. **J** Tunel staining results of lung tissue in in situ A549 tumors in BALB/C-nu mice(100×). **K** Immunohistochemical staining of ORI@HA-cys-TOS micelles using CD31 antibody in A549 and HePG2 tumor vessels (100×). **L** The quantitative analysis based on the imaging in (K) by a software “Image J”. **M** Immunohistochemical staining of ORI@HA-cys-TOS micelles using CD44 antibody in A549 and HePG2 tumors. Nuclei were counterstained with DAPI (100×). (N) The quantitative analysis based on the imaging in ( M) by a software “Image J” (*n* = 3, ***P < 0.001)

Figure 4B and G demonstrates the subcutaneous and in situ A549 tumor model photographs, respectively. The successful establishment of the in situ A549 tumor model in BALB/C-nu mice was confirmed through Ki67 staining, Tunel staining and in situ lung cancer anatomy (Fig. 4E and G). The specific accumulation of ORI@HA-cys-TOS micelles in CD44-positive tumors was investigated simultaneously in subcutaneous and in situ A549 tumor models. Clearly, a gradually increased fluorescence signal were observed in subcutaneous bearing A549 tumor models indicated that significantly more ORI@HA-cys-TOS micelles accumulated into CD44-positive A549 tumors during the whole experiment process (Fig. 4A, C and D). Surprisingly, the specific accumulation of ORI@HA-cys-TOS micelles in in situ A549 tumors was also observed, like the results of cell uptake in vitro (Fig. 3C). The fluorescence intensity of various tissues in the subcutaneous and in situ A549 tumor model after 48 h injection were observed (Fig. 4C and H), and were quantitated (Fig. 4D and I). As showed in Fig. 4A and I, the ORI@HA-cys-TOS micelles exhibited a significantly higher accumulation in both subcutaneous and in situ A549 tumors than normal lung tissue. The results indicated that ORI@HA-cys-TOS micelles could effectively accumulate into the tumor tissue. The specificity could enhance efficacy and minimize damage to non-target organs.

### Immunofluorescence staining analysis

Immunofluorescence staining was performed using FITC-labeled CD31 antibody to observe the distribution of ORI@HA-cys-TOS micelles in A549 and HePG2 tumor vessels. Results from Fig. 4K and L demonstrated a greater degree of overlap between the fluorescence of HA-cys-TOS carriers and CD31 vascular fluorescence in comparison to HepG2 tumors. This indicated that ORI@HA-cys-TOS micelles could target the A549 tumors blood vessels. To further observe the specific binding of CD44 receptor and ORI@HA-cys-TOS micelles in tumor tissues, fluorescence staining was conducted using FITC-labeled CD44 antibodies. As shown in Fig. 4M and N, significantly increased ORI@HA-cys-TOS accumulated in CD44 over-expressed A549 tumors, compared with HepG2 tumors with low CD44 expression (*P* < 0.001). These results showed that ORI@HA-cys-TOS could further extravasate microvessels into deeper tumor tissue and exhibit co-localization with the CD44 receptor in A549 tumor cells, indicating specific binding of micelles with the receptor, which further support the potential of utilizing the interaction between CD44 protein on A549 tumor cells and hyaluronic acid receptor-ligand to enhance drug molecule accumulation at the tumor site, thereby improving treatment efficacy.

### Effect of oridonin on tumor microenvironment

**Fig. 5:**
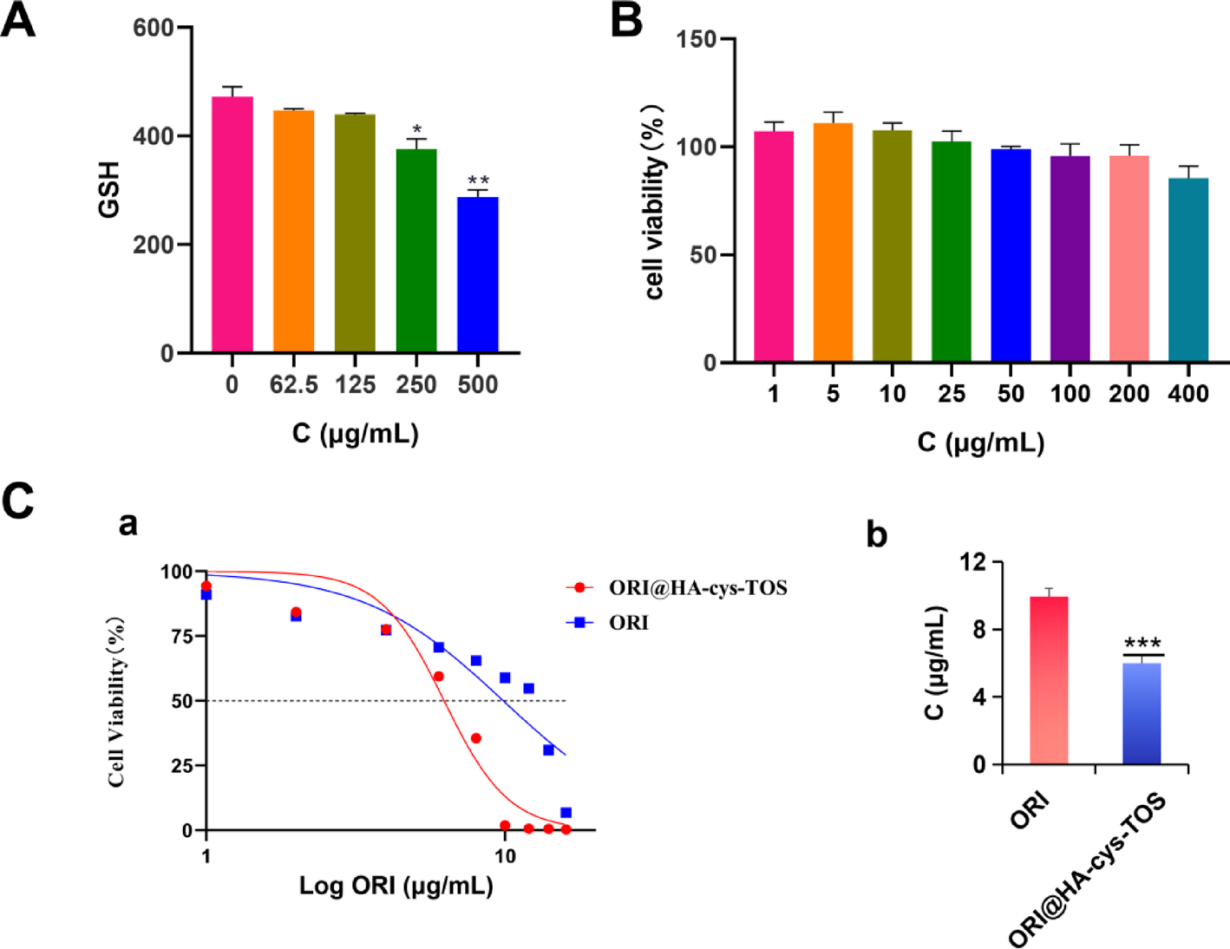
Cytotoxicity study. **A** Consumption effect of Oridonin on GSH. **B** Cytotoxicity of HA-cys-TOS to A549. **C** In vitro cytotoxicity of A549 cells treated with free ORI and ORI@HA-cys-TOS (a). The IC50 value of free ORI and ORI@HA-cys-TOS to A549 (b). All data represented the mean ± standard deviation (*n* = 6, *P < 0.05, **P < 0.01, ***P < 0.001)

Reducing the content of glutathione in tumor cells will cause programmed cell death activated by oxidative stress. Therefore, consuming intracellular GSH can enhance the therapeutic efficacy of drug-loaded micelles. The results of GSH consumption by oridonin are presented in Fig. 5A. Different concentrations of ORI were found to decrease GSH levels, and a high concentration of 250, 500 µg/mL exhibited a significant scavenging effect on GSH (*p* < 0.005). These findings suggest that oridonin’s ability to scavenge glutathione may contribute to its anti-tumor mechanisms.

### Cytotoxicity study

The cytotoxicity of HA-cys-TOS micelles was investigated by adding blank micelles at various concentrations (1, 5, 10, 25, 50, 100, 200, and 400 µg / mL) according to a concentration gradient. Figure 5B demonstrates that the cell survival rate was more than 80%. These results indicated that HA-cys-TOS micelles serve as a relatively safe polymer micelle carrier.

The cell viability of A549 cells was assessed after treatment with free ORI and ORI@HA-cys-TOS for 24 h. As depicted in Fig. 5C, D and E, the IC50 of free oridonin on A549 cells was found to be 9.928 ± 0.494 µg/mL. However, when loaded into micelles, the IC50 increased to 5.997 ± 0.747 µg/mL, resulting in a significant enhancement of the antitumor activity (*p* < 0.001).

### In vivo antitumor activity studies

**Fig. 6:**
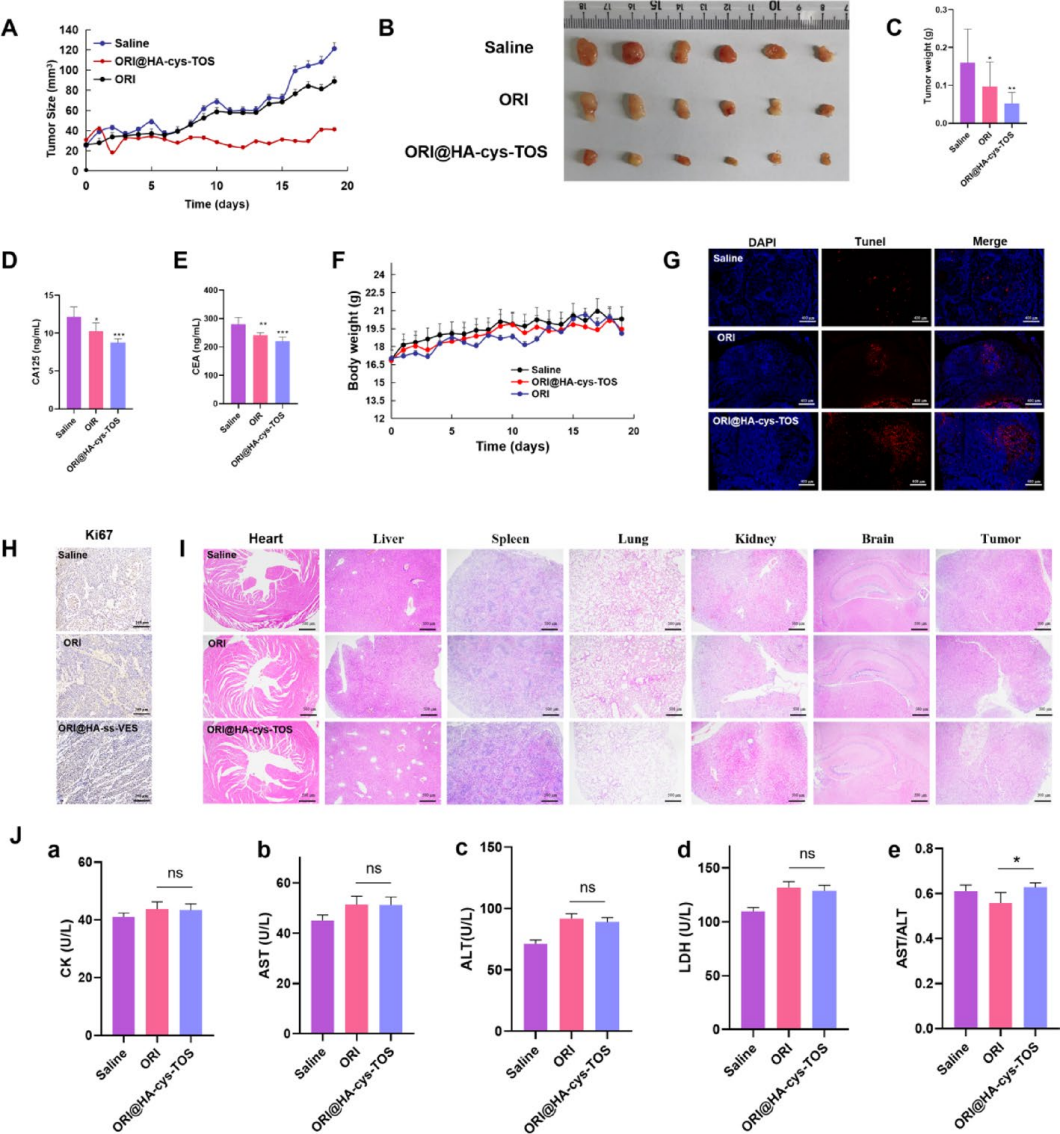
In vivo anti-tumor activity studies. **A** Tumor growth curves for the mice bearing A549 tumors after the intravenous injection of Saline, ORI, ORI@HA-cys-TOS micelles. **B** Representative photographs of tumors in Saline, ORI, ORI@HA-cys-TOS micelles groups after treatment. **C** The average tumor weights in different treatment groups. **D** Contents of CEA in serum. **E** Contents of CA125 in serum. **F** Changes of body weight of mice in different treatment groups. **G** Tunel staining of A549 tumor tissue after 21 days of treatment (100×). **H** Ki67 staining of A549 tumor tissue after 21 days of treatment (100×). **I** H&E staining images of various tissue (40×). **J** CK (a), AST (b), ALT (c), LDH (d) and AST/ALT (e) in serum. All data represented the mean ± standard deviation (*n* = 6, *P < 0.05, **P < 0.01, ***P < 0.001)

The tumor volume growth curve is shown in Fig. 6A. It can be observed that the tumor growth in all groups exhibited an upward trend. However, when comparing the Saline and ORI groups with the ORI@HA-cys-TOS group, it is evident that the ORI@HA-cys-TOS group had a significant inhibitory effect on tumor growth. Following the euthanization of the mice, the tumors were dissected and their weights were measured and analyzed. The obtained results are presented in Fig. 6B and C. The tumor weights for each group were as follows: 0.159 ± 0.089 g in the Saline group, 0.097 ± 0.06 g in the ORI group, and 0.053 ± 0.028 g in the ORI@HA-cys-TOS group. Notably, the tumor weight in the ORI@HA-cys-TOS group exhibited a significant decrease compared to that in the Saline group. The results demonstrate that the drug-loaded micelles exhibited significantly enhanced anti-tumor activity. The changes in body weight of mice are depicted in Fig. 6F. Overall, the body weight of all groups showed a steady increase, and the growth curves of micellar weight for ORI and ORI@HA-cys-TOS closely resembled that of the Saline group. This indicates that the oridonin loaded onto the nano-platform did not have any significant adverse effects on the overall body and exhibited high biological safety. The histopathological changes of each group of mice were evaluated using HE staining. The necrosis and apoptosis of cells in the tumor site were evaluated using Ki67 and TUNEL staining. Figure 6I displays the staining results of ORI@HA-cys-TOS sections in different parts of mice after treatment. The results indicate that the ORI@HA-cys-TOS group did not show any noticeable damage to the heart, liver, spleen, lung, and kidney, compared to the normal saline group. This suggests that ORI@HA-cys-TOS has good biological safety. The results of HE staining revealed that the nuclei and contours of tumor cells in the saline group were clearly visible. However, in the ORI and ORI@HA-cys-TOS groups, the nuclei and contours appeared to be blurred. Additionally, the ORI@HA-cys-TOS group exhibited a higher degree of incomplete and necrotic cells, suggesting that ORI@HA-cys-TOS demonstrated a more effective anti-tumor effect. The Ki67 staining results of the tumor are presented in Fig. 6H. After 21 days of treatment, the tumor cells in the normal saline group exhibited a high positive rate of Ki67 and strong proliferative activity.

However, the Ki67 positive rate of tumor cells in the ORI and ORI@HA-cys-TOS groups seems lower than the normal saline group. Additionally, Tunel staining revealed a higher degree of apoptosis in the ORI@HA-cys-TOS group compared to the other two groups (Fig. 6G), indicating a pronounced inhibition of tumor cell proliferation in the ORI@HA-cys-TOS group.

In this study, we employed an automatic biochemical detection method to assess the safety of drug use by monitoring serum biochemical indexes such as CK, AST, ALT, and LDH. The alterations in ALT and AST levels are commonly utilized in liver pathological examinations, with AST/ALT being a crucial biochemical index. ALT is primarily found in hepatocyte plasma. When there is mild hepatocyte injury, there is an increase in the permeability of the hepatocyte membrane, leading to elevated levels of ALT in the blood. On the other hand, elevated levels of AST in the blood indicate severe damage to hepatocytes. Hence, a higher AST/ALT ratio suggests a more severe hepatocyte injury. The level of serum creatine kinase (CK) is a significant biochemical index that indicates metabolic disorder and tissue damage when abnormally high. Moreover, an increase in serum LDH concentration is indicative of myocardial damage. As shown in Fig. 6J, the ORI and ORI@HA-cys-TOS groups exhibited no damage to the major viscera compared to the saline group, which aligns with the findings from visceral organ pathological staining.

A549 is a human non-small cell adenocarcinoma cell line commonly used in research. Among different types of lung adenocarcinoma, it has the highest positive rate of carcinoembryonic antigen (CEA). The measurement of CEA levels in the blood can accurately indicate the severity of lung cancer, making it a valuable tool for evaluating tumor treatment outcomes. The immune function of patients with lung cancer is impaired due to the influence of autoimmune function. This impairment leads to changes in the concentration of glycoprotein complex CA125 on the cell surface. These changes have significant value for the evaluation and diagnosis of lung cancer. To evaluate the anti-tumor effect of ORI@HA-cys-TOS, the serum of BALB/C-nu nude mice was analyzed for the presence of carcinoembryonic antigen (CEA) and carbohydrate antigen (CA125). The levels of CEA and CA125 tumor markers are depicted in Fig. 6D and E, respectively. The ORI@HA-cys-TOS group demonstrated a significant reduction in serum CEA and CA125 levels (*P* < 0.001), effectively inhibiting the development of A549 lung cancer and exhibiting a favorable anti-tumor effect. These results indicate that the HA-cys-TOS prepared by our team can enhance the anti-tumor effect of free ORI while maintaining good safety, and it also exhibits a strong inhibitory effect on lung cancer.

## Discussion

As a prevalent and deadly malignant tumor worldwide, lung cancer poses a significant risk to human life and well-being [32, 33]. Currently, the primary clinical approach for treating lung cancer involves a combination of surgery, radiotherapy, chemotherapy, and other comprehensive treatments [34, 35]. This approach can effectively manage the advancement of lung cancer to some extent. However, there are still certain challenges that need to be addressed, including low survival rates and diminished quality of life for patients. The high toxicity, side effects, low bioavailability, and drug resistance of most chemotherapeutic drugs contribute to their poor efficacy [36]. Therefore, there is an urgent need to develop more efficient and low-toxicity drugs for treating lung cancer. Oridonin (ORI) is a promising anti-tumor drug that exhibits significant anti-cancer activity [37]. However, its clinical application is severely hindered by its poor solubility and high instability. These limitations pose challenges to the development and utilization of ORI as a potential candidate drug for anti-cancer therapy.

In this study, alpha-tocopherol succinate (ɑ-TOS) with immunomodulatory properties was employed to modify hyaluronic acid (HA), finally developed a ROS-sensitive and CD44 receptor-targeting nano-drug delivery system HA-cys-TOS for the targeted delivery of ORI. The chemical structure of HA-cys-TOS polymer was confirmed by 1 H NMR spectrum (Fig. 1). The synthesized amphiphilic HA-cys-TOS polymer possesses favorable self-assembly performance in aqueous environments, enabling the facile fabrication of uniform micellar nanocarriers (Fig. 2). After ORI After ORI encapsulation, the prepared ORI@HA-cys-TOS micelles presented regular spherical morphology, accompanied by a moderate increase in particle size relative to blank micelles, which verified the successful loading of ORI into the nanocarrier framework (Fig. 2; Table 1). In terms of biological safety, the nanocarrier showed negligible hemolytic activity, confirming superior blood compatibility and great feasibility for intravascular drug delivery. The in vitro drug release results demonstrated that ORI@HA-cys-TOS micelles have good sustained ORI release effect under physiological conditions. Meanwhile, the release of ORI could be effectively triggered by high concentration of GSH, which simulates the high-GSH tumor microenvironment and enables rapid micelle disassembly and drug release (Fig. 2F). This is very much helpful to enhance the therapeutic efficacy of ORI once ORI@HA-cys-TOS micelles were delivered into the tumor tissue. Western blot confirmed high CD44 expression in A549 cells. Combined with the competitive inhibition effect of free HA on cellular internalization, we deduce that elevated CD44 levels are responsible for the enhanced cellular uptake of ORI@HA-cys-TOS micelles (Fig. 3). Consistent with cellular-level findings, ORI@HA-cys-TOS micelles exhibited prominent accumulation in CD44-positive A549 tumors in both subcutaneous and orthotopic tumor model (Fig. 4). These lung cancer mouse models closely recapitulate clinical characteristics, making them a reliable platform to explore lung cancer pathogenesis, assess therapeutic regimens and advance novel drug development. In addition, the enhanced permeability and retention (EPR) effect of solid tumors also contributes to the tumor accumulation of the micelles [30].

As shown in Fig. 4, ORI@HA-cys-TOS micelles exhibited potent CD44 binding and tumor vascular targeting in immunohistochemical assays, which supports CD44-mediated cellular internalization. Combined with the EPR effect for vascular enrichment, the system achieves precise tumor targeting. Such targeting strategy ensures efficient local drug delivery and paves the way for in vivo pharmacodynamic studies of targeted treatment [38–40].

The impact of GSH on the tumor microenvironment was investigated using oridonin treatment. The results showed that ORI could reduce intracellular GSH levels in tumor cells, indicating that the glutathione scavenging effect contributes to the antitumor mechanisms of ORI (Fig. 5A). Further experiments verified that HA-cys-TOS micelles possessed favorable biosafety (Fig. 5B). After loading with ORI, ORI@HA-cys-TOS micelles exerted markedly improved in vitro antitumor activity. In vivo antitumor assays also confirmed that ORI@HA-cys-TOS micelles significantly suppressed the growth of A549 subcutaneous tumor (Fig. 6). This favorable antitumor efficacy could be attributed to multiple factors: the high tumor-specific accumulation of ORI@HA-cys-TOS micelles, enhanced cellular uptake of ORI mediated by HA-cys-TOS carriers, selective cytotoxicity of released ORI in the tumor redox-responsive microenvironment, and immunomodulatory effects of α-tocopherol succinate [41]. As a result, this targeted synergistic and efficient therapy inhibits tumor growth and reduces the progression of lung cancer.

## Conclusion

In this work, to enhance the solubility and stability of oridonin and explore its potential clinical application, ORI@HA-cys-TOS was developed, offering potential CD44 receptor-targeting, redox responsiveness and immunoregulatory effects for the optimized targeted lung cancer therapy. Our data demonstrated the feasibility of increasing the accumulation of the ORI@HA-cys-TOS micelles into CD44-positive tumors by the interaction between HA on the nanoparticles and CD44 receptor on tumor cells. This targeted accumulation was confirmed through in vivo subcutaneous and in situ tumor models, as well as dual tumor-cell co-culture strategy in vitro. ORI@HA-cys-TOS micelles exhibited significantly stronger antitumor efficacy against CD44-positive A549 tumors, effectively inhibiting tumor growth and slowing lung cancer progression while maintaining a high level of biosafety. This enhanced efficacy is likely due to the increased accumulation of the ORI@HA-cys-TOS micelles mediated by CD44 receptor in CD44-positive tumors. In conclusion, the ORI@HA-cys-TOS demonstrated promising tumor-targeting capabilities and high biosafety. These findings support the formulation as a reliable preclinical therapeutic candidate, providing new perspectives for the development of novel ORI-based antitumor strategies for lung cancer treatment.

### Limitations

Despite the enhanced efficacy could be attributed to CD44-mediated accumulation, underlying molecular mechanisms remain unclear. Specific redox-responsive pathways in the tumor microenvironment and detailed immunoregulatory effects (e.g., on immune subsets, cytokines) were not systematically studied, restricting comprehensive understanding of its therapeutic potential. Additionally, the pharmacokinetic and pharmacodynamic profiles have not been verified in large animal models or human-derived organoids, which warrants further investigation.

## Supplementary Information

Below is the link to the electronic supplementary material.

Supplementary Material 1

## Data Availability

All relevant data generated or analyzed during this study are included in this published article (and its supplementary information files). No sequence data, genomic data, or other mandated deposition types (e.g., protein/DNA/RNA sequences, macromolecular structures) were generated. Raw experimental data are available from the corresponding author upon reasonable request.
