# Supplementary material for: Oridonin loaded amphiphilic hyaluronic acid polymeric micelle with tunable redox sensitive property for CD44 targeted lung cancer therapy
Source: Discov Nano. 2026 Jul 10;21(1):334. doi: 10.1186/s11671-026-04798-x (PMC13350622; doi:10.1186/s11671-026-04798-x)
Supplement: Supplementary file 1 — Supplementary Material 1 [file 11671_2026_4798_MOESM1_ESM.docx]

****Supplementary Material****

This file contains supplementary Fig. S1, Fig. S2, Tab. S1, Tab. S2, Tab. S3 and Tab. S4. The results showed that we established content determination method was demonstrated to be accurate and reliable.

****1. Establishment of the Oridonin (ORI) Content Determination Method****

****1.1 Specificity Investigation****

As shown in Fig. S1, the retention time of ORI was approximately 10 min. The solvent peak was consistent with that of the methanol solution. No interfering peaks were observed at the corresponding retention time in the chromatogram of the blank micelle negative control (HA-ss-TOS). These results indicate good separation of ORI and demonstrate the absence of interference from the mobile phase or the blank matrix under the analytical conditions.


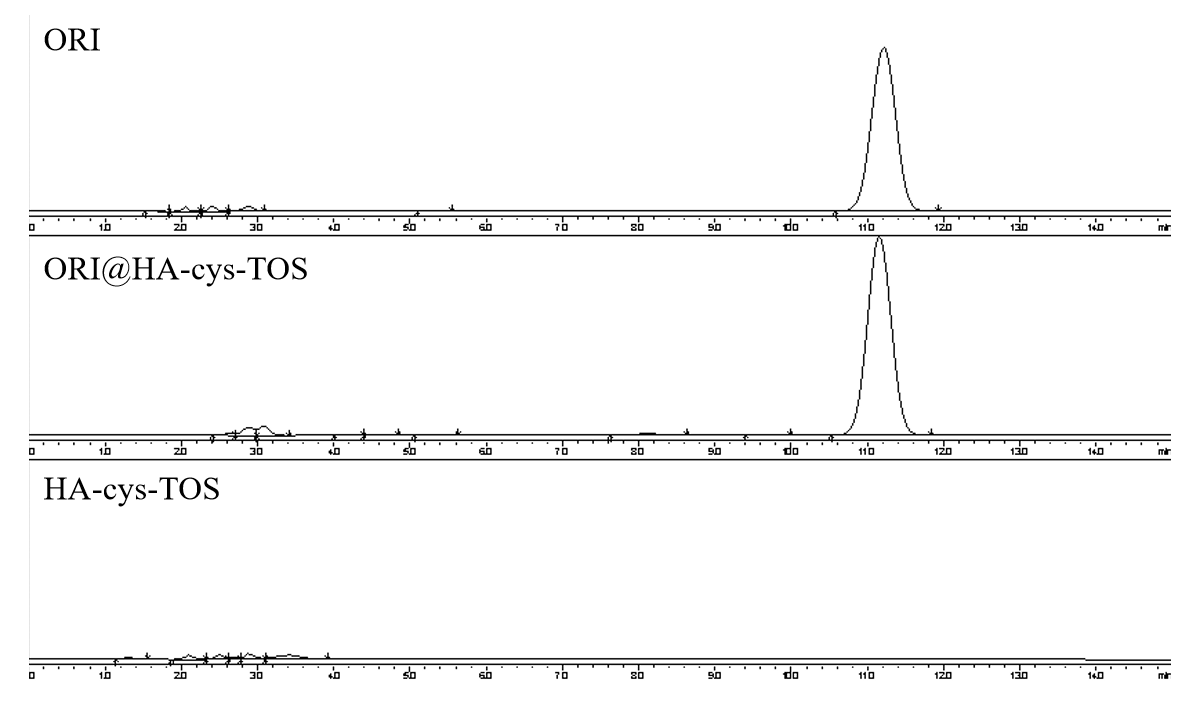

**Fig. S1.** Chromatogram demonstrating the specificity of the ORI assay.

****1.2 Linearity Investigation Results****

The linear regression equation for ORI was y = 29889x - 1161.1 (R² = 0.9997), as depicted in Fig.S2. This indicates an excellent linear relationship between the peak area and ORI concentration within the range of 1–100 µg/mL.

Fig. S2. Linear relationship diagram of oridonin


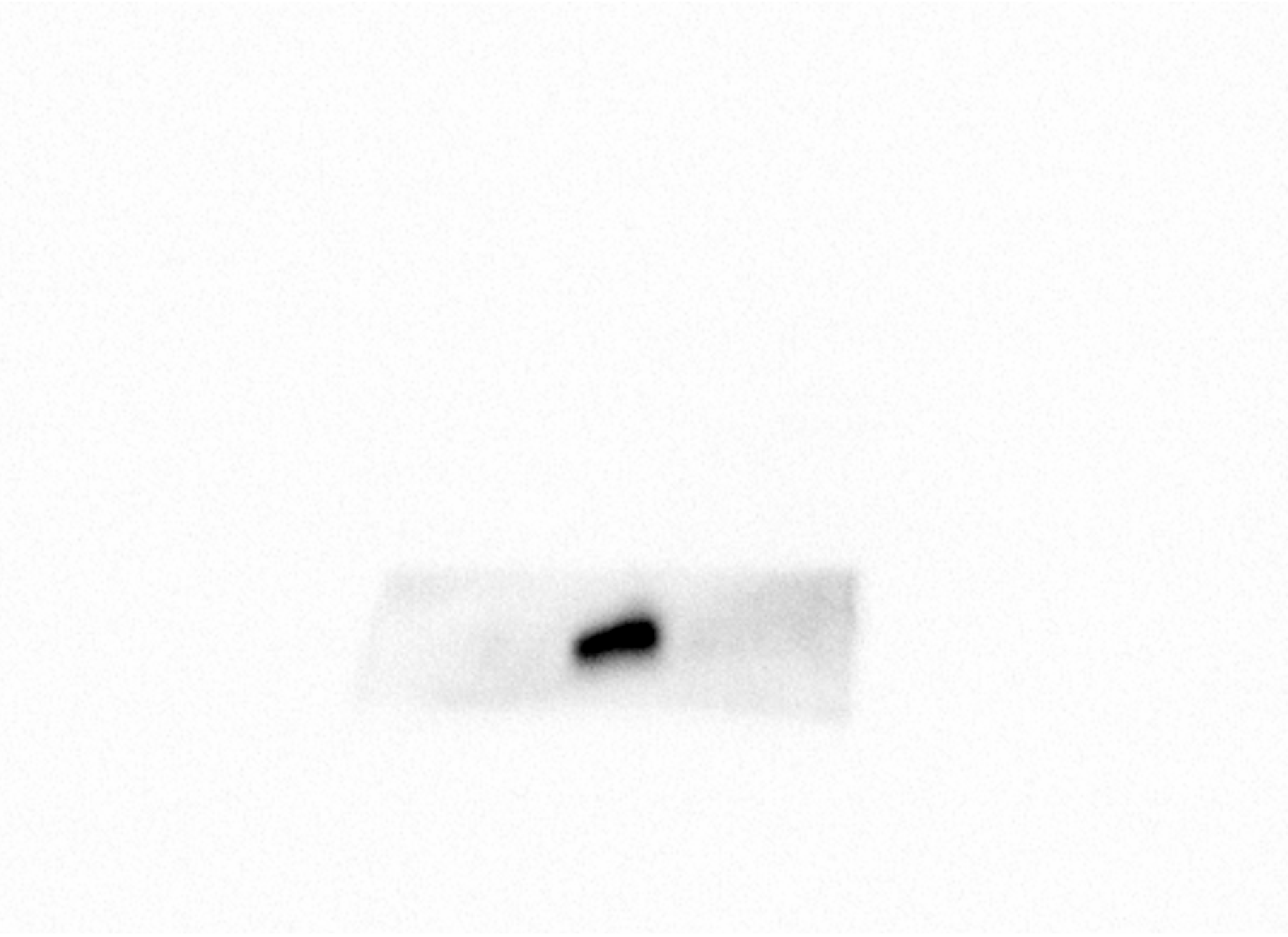

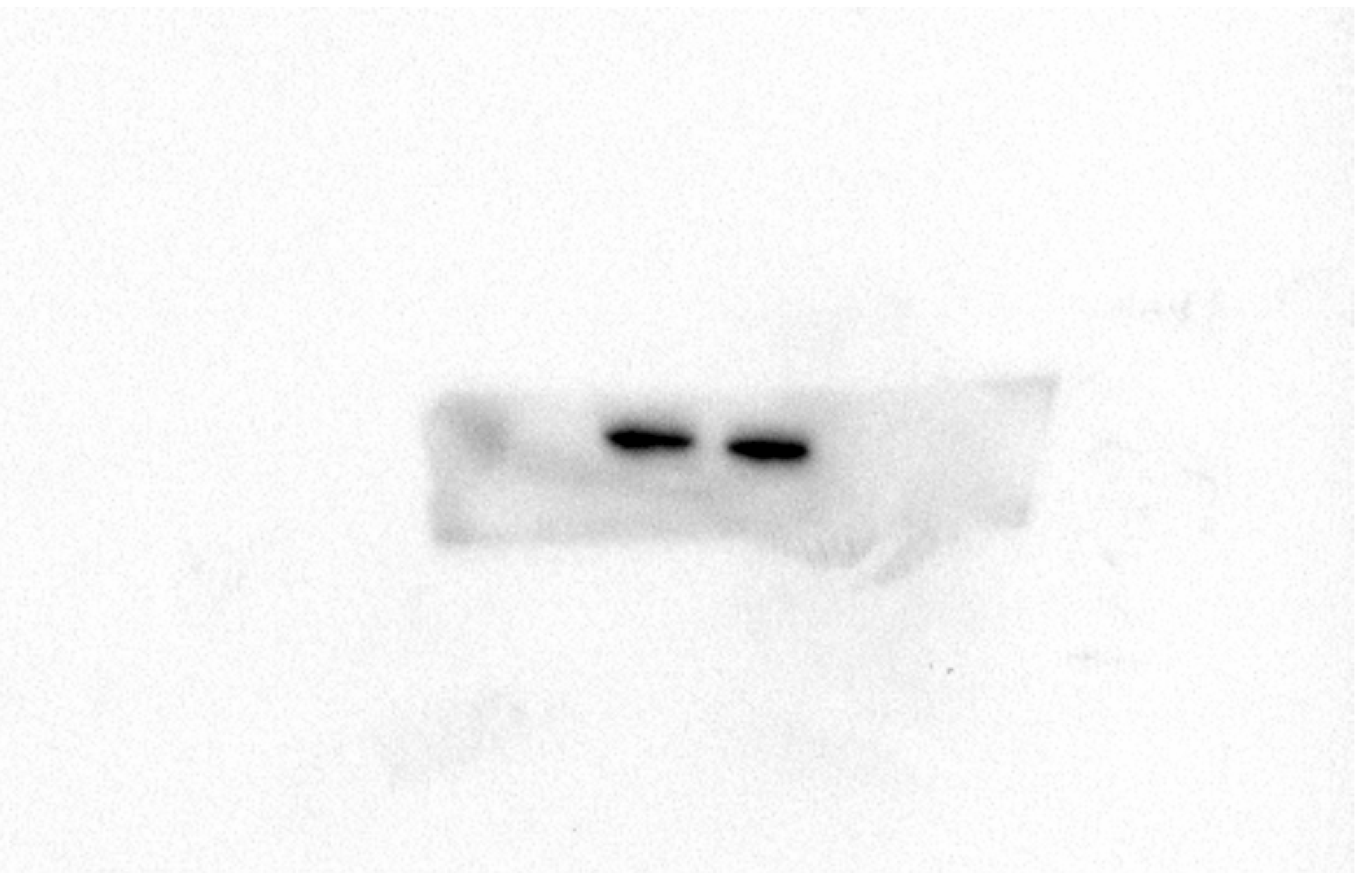


Figure S3.The full original blot membranes of Figure 3E

****1.3 Precision Results****

The relative standard deviation (RSD) values for precision of ORI were all below 3% (Tab.S1), confirms the satisfactory precision of the analytical system.
 Tab. S1 the precision of oridonin

| within-day | | - Daytime | |
| --- | --- | --- | --- |
| Concentration（µg/mL） | RSD% | Concentration（µg/mL） | RSD% |
| 117.721±0.545 | 0.46% | 118.706±0.987 | 0.83% |
| 77.224±0.492 | 0.64% | 77.432±0.320 | 0.41% |
| 36.964±0.609 | 1.65% | 37.208±0.442 | 1.19% |

****1.4 Repeatability Results****

The RSD (%) for ORI content determined was less than 3% (Tab. S2), which demonstrates good repeatability of the analytical method.

Tab. S2 repeatability of Oridonin

| No | 1 | 2 | 3 | 4 | 5 | 6 | RSD（%） |
| --- | --- | --- | --- | --- | --- | --- | --- |
| Concentration  （µg/mL） | 3.335 | 3.444 | 3.428 | 3.472 | 3.443 | 3.451 | 1.42 |

****1.5 Stability Results****

The RSD (%) for ORI content assessed was below 2% (Tab. S3), which confirms the stability of the samples within the tested 48-hour period.
 Tab. 3 Stability results of oridonin

| Time（h） | 0 | 1 | 2 | 4 | 8 | 16 | 24 | 48 | RSD（%） |
| --- | --- | --- | --- | --- | --- | --- | --- | --- | --- |
| Concentration  （µg/mL） | 117.564 | 119.138 | 117.850 | 116.356 | 117.456 | 119.601 | 121.110 | 117.964 | 1.26 |

****1.6 Recovery Results****

Recovery rates determined ranged from 95% to 105%, with an RSD (%) below 3 (Tab. S4), which indicate satisfactory recovery using the developed method.
 Tab. S4 results of recovery of ORI by adding samples

| Sample Weight  (µg） | Addition  (µg) | Measured amount  (µg) | Recovery rate (%) | Average recovery rate  （%） | RSD（%） |
| --- | --- | --- | --- | --- | --- |
| 49.507 | 25.845 | 75.437 | 100.11% |  |  |
| 49.507 | 25.845 | 75.898 | 100.72% | 99.79% | 1.14% |
| 49.507 | 25.845 | 74.244 | 98.53% |  |  |
| 49.507 | 50.516 | 101.442 | 101.42% |  |  |
| 49.507 | 50.516 | 102.396 | 102.37% | 101.61% | 0.67% |
| 49.507 | 50.516 | 101.065 | 101.04% |  |  |
| 49.507 | 75.187 | 128.265 | 102.86% |  |  |
| 49.507 | 75.187 | 129.243 | 103.65% | 102.88% | 0.74% |
| 49.507 | 75.187 | 127.337 | 102.12% |  |  |
